# Exogenous Trilobatin Enhances Flavonoid Content in Purple Rice Grains and Affects the Flavonoid Biosynthesis Pathway

**DOI:** 10.3390/plants13233389

**Published:** 2024-12-03

**Authors:** Qiangqiang Xiong, Han Wu, Runnan Wang, Siqi Tang, Haihua Luo

**Affiliations:** 1 Research Institute of Rice Industrial Engineering Technology, Yangzhou University, Yangzhou 225009, China; 2 Jiangsu Key Laboratory of Crop Genetics and Physiology/Jiangsu Key Laboratory of Crop Cultivation and Physiology, Agricultural College of Yangzhou University, Yangzhou 225009, China; 3 Jiangsu Co-Innovation Center for Modern Production Technology of Grain Crops, Yangzhou University, Yangzhou 225009, China; 4 College of Pharmacy, Gannan Medical University, Ganzhou 341000, China; 5 Guangdong Provincial Key Laboratory of Conservation and Precision Utilization of Characteristic Agricultural Resources in Mountainous Areas, Jiaying University, Meizhou 514015, China

**Keywords:** trilobatin, purple rice, metabolomics, flavonoid biosynthesis, total antioxidant capacity

## Abstract

Antioxidant activity and flavonoid content are important characteristics of colored rice grains. Previously, we obtained a preliminary understanding of the metabolic markers of antioxidant activity, namely, phlorizin and trilobatin, in different colored rice varieties and purple rice grains at different growth stages, but the mechanisms associated with these markers have not yet been confirmed. In this study, purple rice was selected as the experimental material, and clover extract was applied during the grain-filling stage to explore the impact of clover extract on the total antioxidant capacity and flavonoid biosynthesis in purple rice grains. The results indicated that the total flavonoid content, total phenolic content, oligomeric proanthocyanidin content, and total antioxidant capacity of purple rice grains treated with an exogenous application of trilobatin (T30) were significantly greater than those of the control (CK). The flavonoids in the T30 and CK groups accounted for 29.81% of the total flavonoids. The phenylpropanoid biosynthesis and flavonoid biosynthesis metabolic pathways were constructed on the basis of the differentially abundant metabolites between the T30 and CK groups. Additionally, 1-O-sinapoyl-beta-D-glucose, coniferaldehyde, 6″-acetylapiin, and kaempferol-3-O-rutinoside were determined to be essential metabolites for trilobatin-mediated flavonoid biosynthesis in purple rice. The correlation network diagram between biochemical indexes and metabolites revealed that 1-O-sinapoyl-beta-D-glucose, coniferaldehyde, 6″-acetylapiin, and kaempferol-3-O-rutinoside were important metabolites. This study provides a scientific basis for improving the nutritional quality of rice grains and understanding the flavonoid biosynthesis pathway.

## 1. Introduction

Rice is among the most important food crops worldwide. As rice originated in China, among other countries, China now possesses rich germplasm resources [1]. Colored rice is an important resource of special rice germplasms and is formed by the deposition of different pigments in the seed coat; the most common colored rice is purple rice [2,3]. Rice is rich in vitamins, trace elements, and polyphenols such as flavonoids and anthocyanins, and has high nutritional value and specific health care functions [4,5]. Anthocyanins are a class of flavonoid pigments synthesized via a secondary metabolic pathway from the amino acid phenylalanine, and they impart purple, red, and blue coloration in plants. This pathway has been studied in detail in a few plant species such as maize, barley, petunia, and antirrhinum. The enzymes and corresponding genes involved in anthocyanin synthesis and pathway regulation have also been identified [6,7]. Polyphenols and flavonoids in purple rice scavenge free radicals and provide antioxidant activities, which can reduce fat and cholesterol intake, improve antioxidant capacity, reduce oxidative damage to arterial wall cells and other components, and prevent cancer [8]. Colored rice has a high content of phenols and flavonoids, a strong reducing ability, and the ability to inhibit breast cancer, melanoma, and oral cancer [9]. Flavonoids have various beneficial effects in humans, and understanding the pathways involved in flavonoid biosynthesis will help elucidate the functions and potential uses of these compounds [10]. Anthocyanins are antioxidants that are used as natural coloring agents and are beneficial for human health. Anthocyanins can reduce the levels of serum creatinine, blood urea nitrogen (BUN), renal xanthine oxidase (XOD), malondialdehyde (MDA), and nitric oxide (NO) [11]. Proanthocyanidins in colored rice can inhibit the expression of proteins such as intercellular adhesion molecule-1 and interleukin-6, which are related to extracellular matrix degradation; a previous study showed that proanthocyanidins reduced the migration and invasion of MDA-MB-231 human breast cancer cells [12]. Polyphenols such as flavonoids and anthocyanins are widely used in health foods, medicines, and beauty products because of their unique antioxidant properties [13,14]. Colored rice polyphenols can bind to the active center of pancreatic α-amylase through hydrogen bonds, thus inhibiting the activity of pancreatic α-amylase and reducing the effect of blood sugar [15]. Trilobatin is a member of the dihydrochalcone family and has antioxidant, anti-inflammatory, and antidiabetic activities. Trilobatin is a potent α-glucosidase inhibitor that helps ameliorate type 2 diabetes mellitus [16]. Researchers reported that eating purple rice bread significantly improved the postprandial blood sugar and antioxidant status of healthy volunteers [17]. Moreover, when healthy volunteers ate yogurt that contained 0.25% purple rice anthocyanin, postprandial glucose levels and the antioxidant capacity of plasma were significantly inhibited [18]. Previously, we identified metabolic markers of antioxidant activity, namely, phlorizin and trilobatin, in different colored rice varieties and purple rice grains at different growth stages [2,19]. However, the changes in oxidation resistance and synthesis of metabolic pathways in rice grains caused by the exogenous spraying of phlorizin and trilobatin have not been tested in a field setting. Whether exogenous spraying can improve antioxidant activity and flavonoid compounds needs to be further verified. In addition, trilobatin is an important substance in flavonoid biosynthesis [20]. Therefore, further examination of the pathway of flavonoid biosynthesis mediated by metabolic markers in purple rice grains is highly important for product development when purple rice grains are used as raw materials. In this study, the purple rice variety Yangzinuo 1 hao, which was independently bred by the Institute of Rice Industry Engineering and Technology of Yangzhou University, was selected, and the metabolic markers of antioxidant characteristics in its grains were identified by spraying the exogenous compounds phlorizin and trilobatin. Furthermore, nontargeted metabonomic and biochemical data were integrated to clarify the pathways involved in flavonoid biosynthesis mediated by metabolic markers. A multifactor correlation network diagram was subsequently constructed, providing basic data for the cultivation of high-antioxidant rice varieties and for metabolic engineering.

## 2. Materials and Methods

### 2.1. Plant Material and Growth Conditions

The experimental material was Yangzinuo 1 hao (purple rice), which was independently bred by the Institute of Rice Industry Engineering and Technology of Yangzhou University. The rice plants were sown on 16 May 2023, and were raised with a blanket. After 25 days, the plants were transplanted; 4 plants were planted in each well, and the spacing between rows and between plants was 30 cm and 12 cm, respectively. The rice was planted with 3 replicates in Shatou Base, Guangling District, Yangzhou University, Yangzhou city, Jiangsu Province. A total of 300 kg hm^−2^ of pure nitrogen was applied, and the ratio of base fertilizer–tiller fertilizer–panicle fertilizer was 5:3:2. Disease, pest, and weed control was implemented according to the requirements for conventional high-yield cultivation of rice. The Yangzinuo 1 hao plants were sprayed with 30 mg/L phlorizin or trilobatin at the grain-filling stage (21 October 2023) [21,22] (designated P30 and T30, respectively), and the control (CK) was sprayed with only water.

### 2.2. Sample Collection

The rice plants were harvested on 1 November 2023. The rice grains were collected, transported to the laboratory, peeled with tweezers, placed in a freezing tube, and quickly frozen with liquid nitrogen; this process was repeated four times (each replicate comprised a mixed sample of six rice grains). Finally, the samples were stored in a low-temperature freezer at −20 °C.

### 2.3. Determination of Physiological and Biochemical Indexes

The total flavonoid (FD) content was determined using a plant flavonoid test kit, and the instrument method used was visible spectrophotometry. The content of oligoanthocyanins (OPC) was determined using a plant anthocyanin test kit, and the instrument method used was visible spectrophotometry. The total phenolic (TP) content was determined using a plant total phenolic (TP) test kit, and the instrument method used was visible spectrophotometry. Total antioxidant capacity (ABTS method) based on the 2,2-diazo-bis (3-ethylbenzothiazole-6-sulfonic acid) (ABTS) method was used, and the instrument method used was the enzyme-linked immunosorbent assay. The 2,2-diphenyl-1-picrylhydrazyl (DPPH) method uses total antioxidant capacity (DPPH method), and the instrument method used was the enzyme-linked immunosorbent assay. The ferric ion reducing antioxidant power (FRAP) method uses total antioxidant capacity (FRAP method), and the instrument method used was the enzyme-linked immunosorbent assay. These determination methods were performed using biochemical assay kits (Suzhou Michy Biomedical Technology Co., Ltd., Suzhou, China), with each treatment repeated three times. Detailed plans for measuring all physiological and biochemical indicators can be obtained online (http://www.michybio.com).

### 2.4. Detection of Grain Metabolites

Fifty milligrams of each rice grain sample was placed in a 2 mL centrifuge tube, and grinding beads 6 mm in diameter were added. The sample was extracted with 400 μL of an extraction mixture (methanol–water = 4:1 (*v*:*v*)) containing 0.02 mg mL^−1^ internal standard (L-2-chlorophenylalanine) to obtain the grain metabolites. The sample mixture was ground in a frozen tissue grinder for 6 min (−10 °C, 50 Hz) and subsequently subjected to ultrasonication at a low temperature for 30 min (5 °C, 40 kHz). The sample was incubated at −20 °C for 30 min and centrifuged for 15 min (4 °C, 13,000× *g*), after which the supernatant was transferred to an injection vial with an internal cannula for computer analysis. The metabolites were detected according to the methods of Xiong et al. [23]. The detection of metabolites in this study was carried out by Shanghai Majorbio Biopharm Technology Co., Ltd. Metabolite data were analyzed using the major Bioconductor platform (https://cloud.majorbio.com). Four biological replicates were performed for each treatment. The data were analyzed according to the methods of Ren et al. [24].

### 2.5. Statistical Analysis of Biochemical Indexes

WPS 2021 (Jinshan Office (Beijing) Co., Ltd., Beijing, China) software was used to calculate average values, create figures, and process biochemical data. The Statistical Package for the Social Sciences (SPSS), version 18.0 (International Business Machines Corporation, Armonk, NY, USA) was used to analyze the variance in the grain biochemical data, and Adobe Illustrator CS6 (Adobe Systems Incorporated, San Jose, CA, USA) was used to construct the graphs. All the metabolite variables were scaled to unit variance prior to principal component analysis (PCA) and were scaled by Pareto scaling prior to conducting orthogonal partial least squares-discriminant analysis (OPLS-DA). Multivariate statistical analysis was performed using the ropls R package (version 1.6.2, http://bioconductor.org/packages/release/bioc/html/ropls.html, accessed on 22 May 2024) from Bioconductor on the Majorbio Cloud Platform (https://cloud.majorbio.com). *p* values were estimated with the paired Student’s *t*-test for single-dimensional statistical analysis.

## 3. Results

### 3.1. Biochemical Indicator Analysis

**Figure 1:**
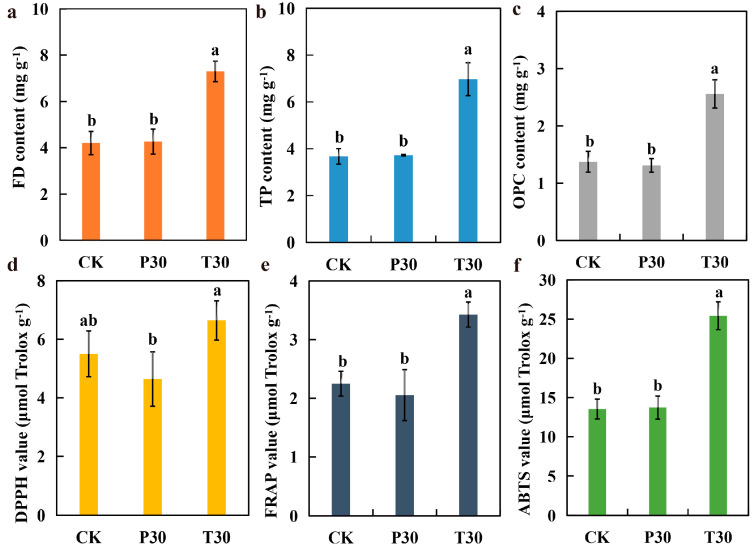
Differences in flavonoid (FD) content and total antioxidant capacity of Yangzinuo 1 hao grains sprayed with exogenous phlorizin and trilobatin. (**a**) FD content; (**b**) total phenolic (TP) content; (**c**) oligomeric proanthocyanidin (OPC) content; (**d**) total antioxidant capacity (DPPH method); (**e**) total antioxidant capacity (FRAP method); (**f**) total antioxidant capacity (ABTS method). Different lowercase letters represent significance at the *p* = 0.05 level. The data are shown as the means + s.e.m.s (n = 3).

To determine the changes in FD content after root bark glycoside and clover extract were sprayed during the grain filling process of purple rice, we constructed histograms of the FD content (Figure 1a), TP content (Figure 1b), OPC content (Figure 1c), total antioxidant capacity (DPPH method) (Figure 1d), total antioxidant capacity (FRAP method) (Figure 1e), and total antioxidant capacity (ABTS method) (Figure 1f) after purple rice grains were sprayed with phlorizin and trilobatin (Figure 1). The FD content of purple rice grains in the T30 treatment was significantly greater than those in the CK and P30 treatments, with increases of 73.52% and 71.18%, respectively. Similarly, the TP content of T30 was significantly greater than those of CK and P30, with increases of 89.49% and 86.96%, respectively. In terms of the OPC content, T30 also presented significant advantages, with values 86.33% and 95.25% greater than those of CK and P30, respectively. In the evaluation of antioxidant performance, the DPPH value of T30 was significantly greater than that of CK, with an increase of 20.72%. Furthermore, the FRAP and ABTS values of T30 were significantly greater than those of CK and P30, with FRAP values 52.35% and 66.83% greater than those of CK and P30, respectively, and ABTS values 87.94% and 75.00% greater than those of CK and P30. In summary, the FD content and total antioxidant capacity of the T30 treatment were significantly greater than those of the CK and P30 treatments. These results indicate that the exogenous application of trilobatin can effectively increase the FD content and total antioxidant capacity of purple rice grains.

### 3.2. Metabolic Spectrum Analysis

**Figure 2:**
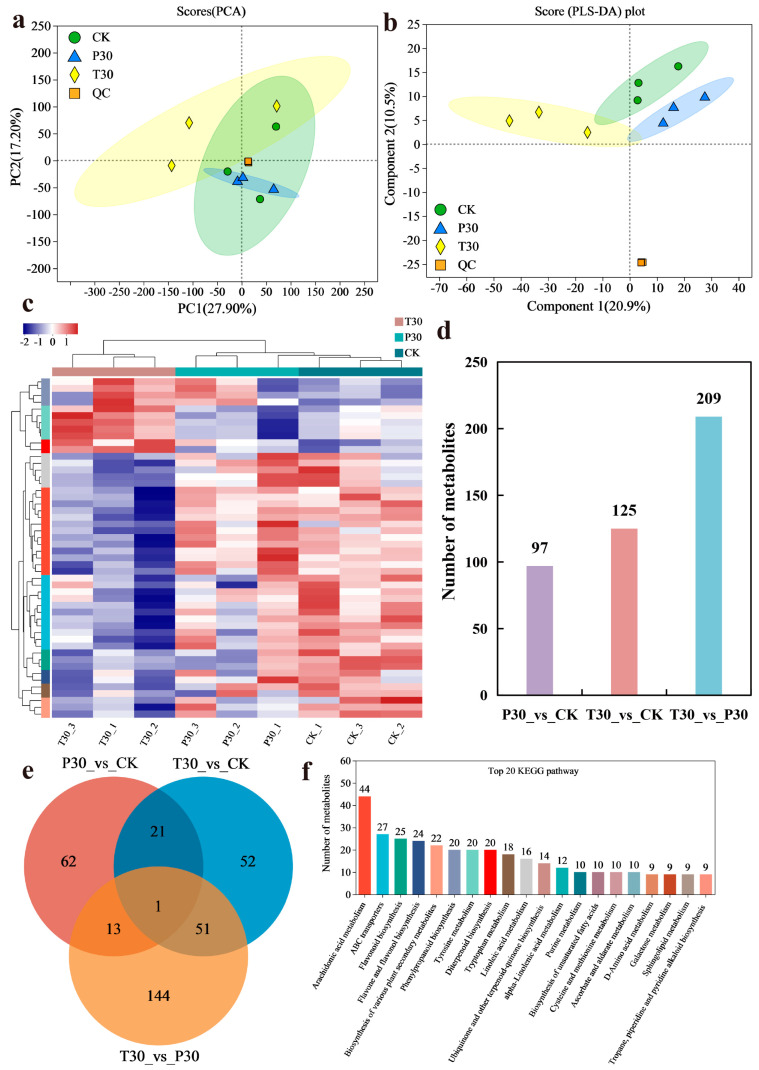
Metabolic spectrum information. (**a**) PCA scores; (**b**) PLS-DA scores; (**c**) metabolite clustering thermogram; (**d**) differentially abundant metabolites in each comparison group; (**e**) Venn diagram of metabolites from different treatments; (**f**) top 20 KEGG pathways. The data are shown as the means + s.e.m.s (n = 4).

Principal component (PC) analysis of the samples (including quality control samples) preliminarily revealed the overall metabolic differences between samples in each group and the within-group variation between samples. The PCA score plot revealed that samples sprayed with the same exogenous substance were closely related, and samples sprayed with different exogenous substances were significantly different. As shown in the PCA score chart, the contribution rate of PC1 was 27.90%, that of PC2 was 17.20%, and the sum of the two principal components was 45.1% (Figure 2a). Partial least squares-discriminant analysis (PLS-DA) further revealed that component 1 explained 20.9% of the variation and component 2 explained 10.5% of the variation (Figure 2b). Significant differences were observed in the metabolic components of the samples due to different treatments. The metabolite accumulation patterns of the rice samples due to different treatments also differed (Figure 2c), which was consistent with the PCA results. Ninety-seven differentially abundant metabolites (DMs) in grains were identified between P30 and CK (Figure 2d; Table S1), 125 DMs were identified between T30 and CK (Figure 2d; Table S2), and 209 DMs were identified between T30 and P30 (Figure 2d; Table S3). The results of the different treatments are visually displayed in a Venn diagram (Figure 2e). The identified DMs mainly participate in flavonoid biosynthesis and flavone and flavonol biosynthesis (Figure 2f).

### 3.3. Classification Statistics of the Compounds

**Figure 3:**
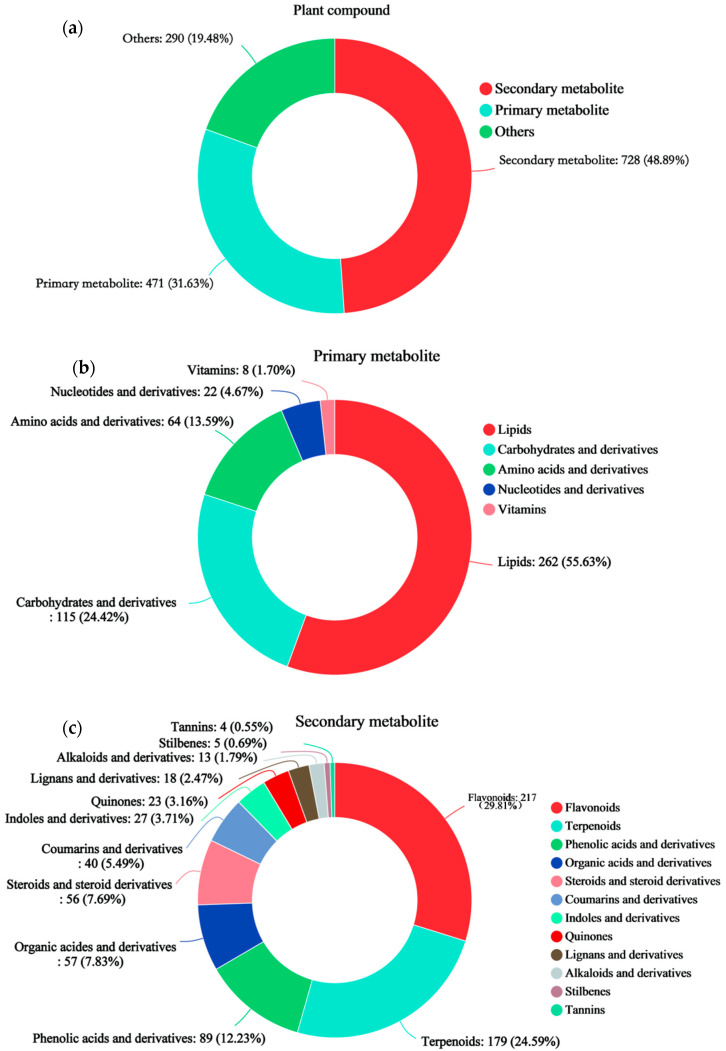
Statistical analysis of compound classes. The type and percentage of metabolites in each class are displayed from high to low according to the number of metabolites for each selected plant compound (primary metabolite or secondary metabolite). The different colors in each pie chart represent different classes, and the area of each section represents the relative proportion of metabolites in that class. (**a**) Plant compounds. (**b**) Primary metabolites. (**c**) Secondary metabolites. The data are shown as the means + s.e.m.s (n = 4).

The identified metabolites were classified and counted. Secondary metabolites accounted for 48.89% of all metabolites (Figure 3a), and primary metabolites accounted for 31.63% (Figure 3a). Other metabolites accounted for 19.48% of the total (Figure 3a). The primary metabolites included lipids, carbohydrates and their derivatives, amino acids and their derivatives, nucleotides and their derivatives, and vitamins (Figure 3b). Among these metabolites, lipids accounted for 55.63%; carbohydrates and their derivatives accounted for 24.42%; amino acids and their derivatives accounted for 13.59%; nucleotides and their derivatives accounted for 4.67%; and vitamins accounted for 1.70%. The secondary metabolites included flavonoids, terpenoids, phenolic acids and their derivatives, organic acids and their derivatives, steroids and their derivatives, coumarins and their derivatives, indoles and their derivatives, quinones, lignans and their derivatives, alkaloids and their derivatives, stilbenes, and tannins (Figure 3c). Among these metabolites, flavonoids accounted for 29.81%; terpenoids accounted for 24.59%; phenolic acids and their derivatives accounted for 12.23%; organic acids and their derivatives accounted for 7.83%; steroids and their derivatives accounted for 7.69%; coumarins and their derivatives accounted for 5.49%; indoles and their derivatives accounted for 3.71%; quinones accounted for 3.16%; lignans and their derivatives accounted for 2.47%; alkaloids and their derivatives accounted for 1.79%; stilbenes accounted for 0.69%; and tannins accounted for 0.55%.

### 3.4. Metabolic Pathways

**Figure 4:**
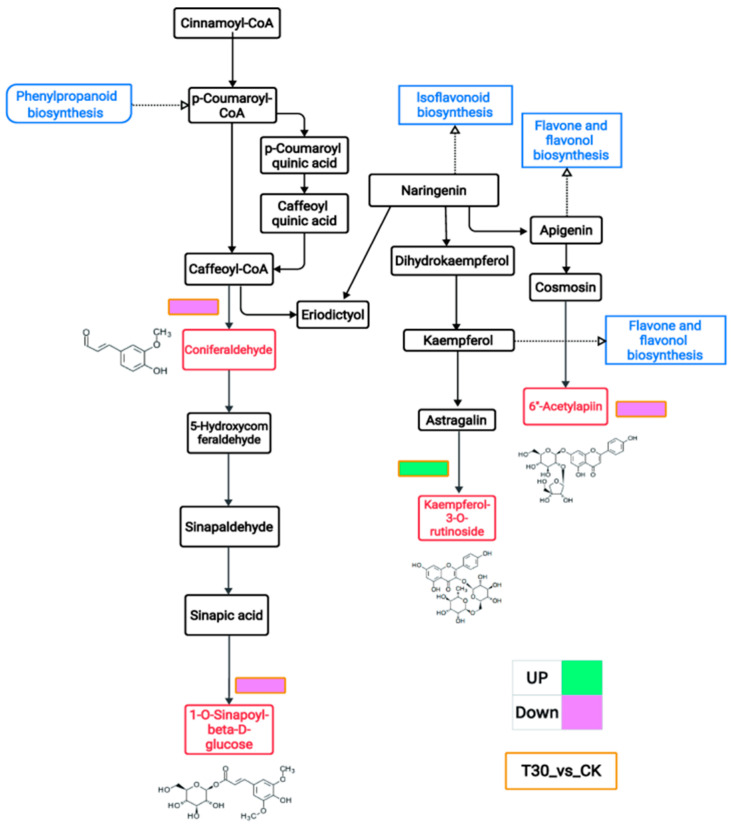
Trilobatin mediates phenylpropanoid biosynthesis and flavonoid biosynthesis in the T30 vs. CK comparison group. The metabolic pathways are shown in blue boxes. The red boxes represent the differentially abundant metabolites (DMs). Green represents upward adjustment, and pink represents downward adjustment.

**Figure 5:**
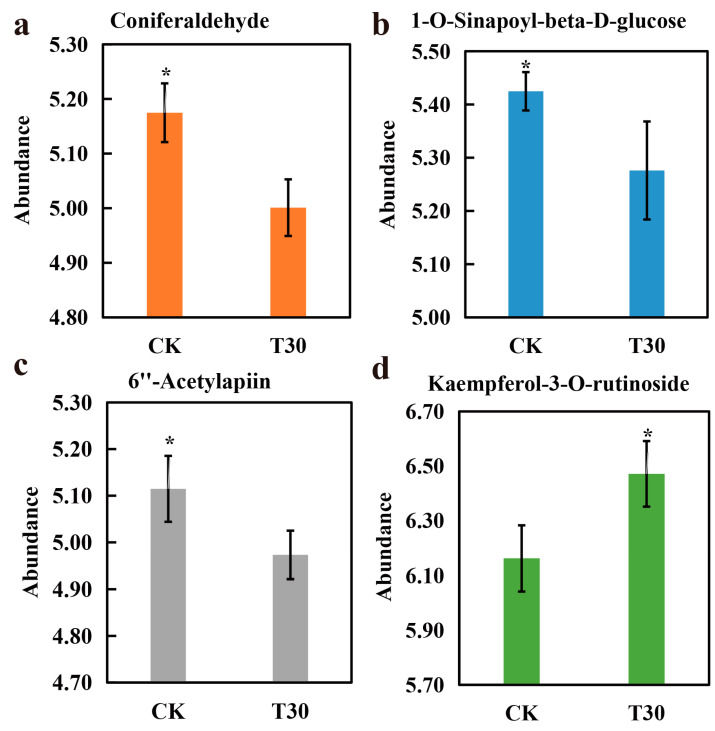
Abundances of four metabolites in T30 and CK. (**a**) Abundance of coniferaldehyde; (**b**) abundance of 1-O-sinapoyl-beta-D-glucose; (**c**) abundance of 6″-acetylapiin; (**d**) abundance of kaempferol-3-O-rutinoside. * represents a *p* value of 0.05. The data are shown as the means + s.e.m.s (n = 4).

Exogenous spraying of trilobatin can significantly improve the total oxidation resistance of Yangzinuo 1 hao grains. In this study, the DMs in the T30 vs. CK comparison group were involved in phenylpropanoid biosynthesis and flavonoid biosynthesis (Figure 4). 1-O-Sinapoyl-beta-D-glucose, coniferaldehyde, 6″-acetylapiin, and kaempferol-3-O-rutinoside were identified as the DMs in the Yangzinuo 1 hao pathway, and the structures of the end products are displayed. Kaempferol-3-O-rutinoside was an upregulated metabolite, and 1-O-sinapoyl-beta-D-glucose, coniferaldehyde, and 6″-acetylapiin were downregulated metabolites. 1-O-Sinapoyl-beta-D-glucose is a phenolic acid and derivative. 6″-Acetylapiin and kaempferol-3-O-rutinoside are flavonoids. Coniferaldehyde is a phenolic compound. A comparison of the abundances of these four metabolites in the T30 and CK groups is shown in Figure 5. The synthesis of the four metabolites is briefly described. Table S4 shows the detailed metabolite information.

### 3.5. Receiver Operating Characteristic (ROC) Analysis

**Figure 6:**
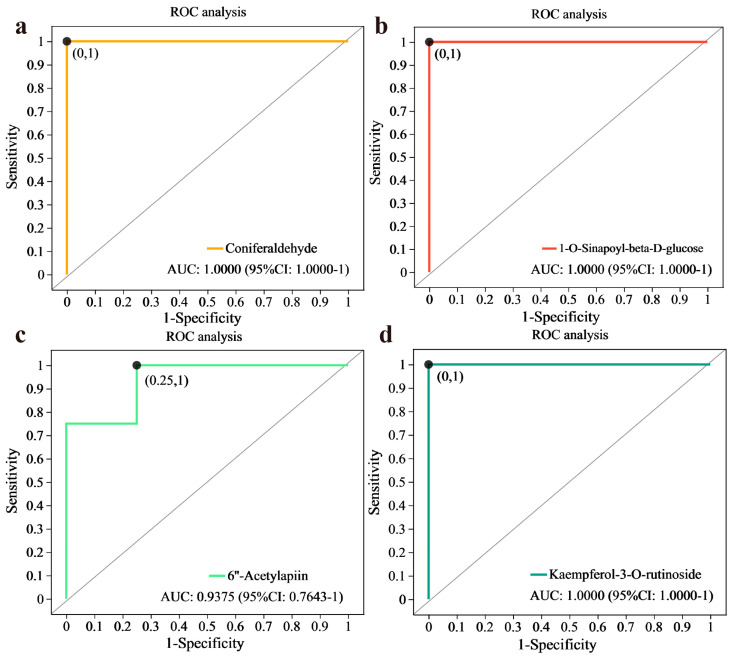
ROC analysis. (**a**) Coniferaldehyde content between T30 and CK; (**b**) 1-O-sinapoyl-beta-D-glucose content between T30 and CK; (**c**) 6″-acetylapiin content between T30 and CK; (**d**) kaempferol-3-O-rutinoside content between T30 and CK.

Receiver operating characteristic (ROC) analysis was performed to analyze the similarity between groups and test the significance of differences between groups. Furthermore, supervised learning was used to perform linear discrimination and classification modeling for groups divided by sample, and a number of biomarker variables that were key to the differences between groups were discovered (Figure 6). When the area under the curve (AUC) is >0.5, a better diagnostic effect is achieved when the AUC is closer to 1. The AUC indicates low accuracy between 0.5 and 0.7, some accuracy between 0.7 and 0.9, and high accuracy above 0.9. The AUCs of coniferaldehyde, 1-O-sinapoyl-beta-D-glucose, and kaempferol-3-O-rutinoside between T30 and CK were 1 and 1, respectively (Figure 6a,b,d). The AUCs of 6″-acetylapiin between T30 and CK were 0.9375 and 1, respectively (Figure 6c).

### 3.6. Correlation Network Analysis

**Figure 7:**
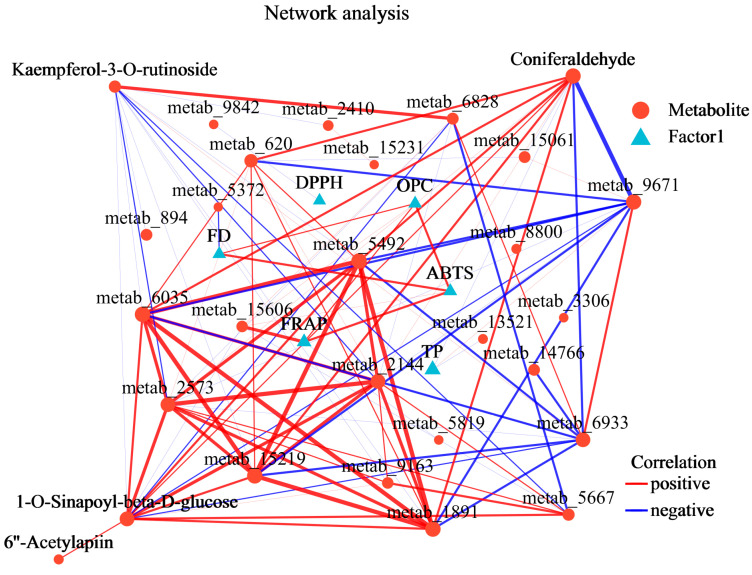
Correlation network diagram. The absolute value of the correlation coefficients between DMs in the T30 and CK groups is greater than 0.9. The multifactor correlation network diagram reflects the correlation between the classification levels of metabolites and between the classification levels of metabolites and species under a certain environmental condition. “Degree” refers to the connection between two metabolites. The size of each node in the figure indicates the degree of the node, and different colors indicate different species. The colors of the connecting lines indicate positive and negative correlations; red indicates a positive correlation, and blue indicates a negative correlation. The thickness of the line indicates the size of the correlation coefficient; the thicker the line is, the greater the correlation between species. The more lines there are, the closer the connections between nodes.

We constructed a correlation network diagram of T30 vs. CK (Figure 7). The metabolites included four end products: kaempferol-3-O-rutinoside, coniferaldehyde, 1-O-sinapoyl-beta-D-glucose, and 6″-acetylapiin. The factors included the following biochemical indexes: FD, DPPH, OPC, ABTS, FRAP, and TP. The correlation coefficients of 1-O-sinapoyl-beta-D-glucose with coniferaldehyde, 6″-acetylapiin, and kaempferol-3-O-rutinoside were 0.929, 0.929, and −0.905, respectively, with 40 degrees of connection for 1-O-sinapoyl-beta-D-glucose, 44 degrees for coniferaldehyde, 5 degrees for 6″-acetylapiin, and 19 degrees for kaempferol-3-O-rutinoside.

## 4. Discussion

Purple rice plants have a high flavonoid content and strong antioxidant capacity [23,25]. Previously, we identified metabolic markers of oxidation resistance, such as phlorizin and trilobatin, in different colored rice varieties and purple rice grains at different growth stages [2,19]. The antioxidant activity of flavonoids is one of their most important forms of biological activity. Flavonoids can clear free radicals through various mechanisms, such as direct reactions with free radicals, activation of the antioxidant enzyme system, and inhibition of oxidase activity [11]. On the basis of the biological activity of trilobatin, the biosynthetic pathways of flavonoids, and preliminary experimental evidence [2,19], we can reasonably speculate that trilobatin may participate in or affect the biosynthesis of flavonoids in some way. Therefore, this study verified the effectiveness of the spraying of phlorizin and trilobatin as metabolic markers of antioxidant activity in purple rice grains. The FD content (Figure 1a), TP content (Figure 1b), OPC content (Figure 1c), total antioxidant capacity (DPPH method) (Figure 1d), total antioxidant capacity (FRAP method) (Figure 1e), and total antioxidant capacity (ABTS method) (Figure 1f) of rice grains treated with exogenous phlorizin were not significantly different from those of the CK treatment group. The FD content (Figure 1a), TP content (Figure 1b), OPC content (Figure 1c), total antioxidant capacity (DPPH method) (Figure 1d), total antioxidant capacity (FRAP method) (Figure 1e), and total antioxidant capacity (ABTS method) (Figure 1f) of the purple rice plants after the exogenous application of trilobatin were significantly greater than those of the CK plants. These findings further demonstrate that trilobatin is an effective metabolic marker of antioxidant activity in purple rice grains. Trilobatin is an antioxidant that can inhibit the production of free radicals and is beneficial for preventing aging and protecting cardiovascular health [26]. The anthocyanin biosynthesis pathway in plants is a branch of the flavonoid biosynthesis pathway that can be divided into two stages: the first stage involves the phenylpropanoid metabolic pathway, and the second stage involves the flavonoid pathway [11,27,28]. In this study, the DMs resulting from the exogenous spraying of phlorizin and trilobatin were involved mainly in flavonoid biosynthesis, flavone and flavonol biosynthesis, and phenylpropanoid biosynthesis (Figure 2f). Flavonoids accounted for the greatest proportion of the secondary metabolites (Figure 3c). This finding also revealed that the exogenous spraying of trilobatin mainly affected the phenylpropanoid biosynthesis and flavonoid biosynthesis pathways. Therefore, we constructed simple metabolic pathways for phenylpropanoid biosynthesis and flavonoid biosynthesis on the basis of the DMs of the T30 vs. CK comparison group (Figure 4). We further found that trilobatin mainly mediated the synthesis of 1-O-sinapoyl-beta-D-glucose, coniferaldehyde, 6″-acetylapiin, and kaempferol-3-O-rutinoside, with increasing and decreasing metabolite contents. 6″-Acetylapiin and kaempferol-3-O-rutinoside are flavonoids (Table S4). Further studies should be performed to determine whether changes in the levels of 6″-acetylapiin and kaempferol-3-O-rutinoside substantially increase the antioxidant capacity of purple rice grains. ROC analysis was performed to analyze the similarity between different groups of samples, test the significance of differences between groups, and explore the biomarkers that distinguish groups [29]. ROC analysis of the T30 and CK groups revealed that the AUC values of 1-O-sinapoyl-beta-D-glucose, coniferaldehyde, 6″-acetylapiin, and kaempferol-3-O-rutinoside were greater than 0.9 (Figure 6), demonstrating high accuracy for these four metabolites. Multiple factor network analysis can be used to calculate the correlation coefficients between characteristic values connecting significantly related characteristic nodes, and the interactions between different variables (metabolites or biochemical indicators) can be represented with lines of different thickness. To simplify complex systems, useful information must be extracted [24]. In this study, a correlation network diagram was constructed, which revealed 40 degrees of connection for 1-O-sinapoyl-beta-D-glucose, 44 degrees for coniferaldehyde, 5 degrees for 6″-acetylapiin, and 19 degrees for kaempferol-3-O-rutinoside (Figure 7). The correlation coefficients of 1-O-sinapoyl-beta-D-glucose with coniferaldehyde, 6″-acetylapiin and kaempferol-3-O-rutinoside were 0.929, 0.929, and −0.905, respectively. These results demonstrated that the correlation network diagrams constructed for four metabolites, flavonoids, TP, OPC, DPPH, FRAP, and ABTS were closely related. The results showed that the metabolites affected by trilobatin are related to biochemical factors to some extent. The levels of flavonoids and phenolic metabolites are closely related to the total antioxidant potential of purple rice grains. These findings indicate that the exogenous spraying of trilobatin can increase the flavonoid content and antioxidant capacity of purple rice grains, as well as alter their flavonoid synthesis pathway. Further studies are needed to determine whether functional rice varieties with a high total antioxidant capacity can be cultivated by targeting metabolites. This study provides reference data for analyzing the flavonoid biosynthesis pathway mediated by trilobatin.

## 5. Conclusions

This study verified that the total flavonoid content and total antioxidant capacity of purple rice grains were significantly improved through the exogenous administration of trilobatin; in addition, the pathway of trilobatin-mediated flavonoid biosynthesis in purple rice grains was further clarified. 1-O-Sinapoyl-beta-D-glucose, coniferaldehyde, 6″-acetylapiin, and kaempferol-3-O-rutinoside were identified as the end products of trilobatin-mediated flavonoid biosynthesis. Furthermore, a correlation network diagram was constructed using the biochemical data and metabolites. This study provides reference data for analyzing the pathway of flavonoid biosynthesis mediated by trilobatin. In the future, advanced biotechnology can be used to target metabolites and cultivate functional rice varieties with high concentrations of total flavonoids and high antioxidant capacity.

## Data Availability

The original contributions presented in the study are included in the article/supplementary material, further inquiries can be directed to the corresponding author.

## Supplementary Materials

The following supporting information can be downloaded from https://www.mdpi.com/article/10.3390/plants13233389/s1. Table S1: DMs between P30 and CK; Table S2: DMs between T30 and CK; Table S3: DMs between T30 and P30; Table S4: Information on four key metabolites involved in the synthesis of flavonoids mediated by T30 vs. CK.
